# Nursing Clinical Teachers' Knowledge, Attitudes, and Practices about Nursing Students Suffering from Workplace Violence in China: A Cross-Sectional Survey

**DOI:** 10.1155/2023/8844919

**Published:** 2023-10-16

**Authors:** Hongwei Chang, Longti Li, Xiaofeng Li, Lifang Zhang, Wen Huang, Hongxia Zhu, Jiao He, Yilan Liu

**Affiliations:** ^1^Department of Nursing, Union Hospital, Tongji Medical College, Huazhong University of Science and Technology, Wuhan 430022, China; ^2^Department of Nursing, Taihe Hospital, Shiyan 442000, China; ^3^Department of Nursing, Second Hospital of Yichang, Yichang 443000, China; ^4^Department of Nursing, Huangshi Central Hospital, Huangshi 435000, China; ^5^Department of Nursing, Wuhan Dongxihu District People Hospital, Wuhan 430040, China; ^6^Department of Nursing, Xiaogan Maternal and Child Health Hospital, Xiaogan 432000, China; ^7^Department of Pediatrics, Union Hospital, Tongji Medical College, Huazhong University of Science and Technology, Wuhan 430022, China

## Abstract

**Aims:**

To measure nursing clinical teachers' knowledge, attitudes, and practices about nursing students (NSs) suffering from workplace violence (WPV) in China.

**Background:**

The nursing clinical teacher is the most important person for NS during the clinical rotation. In contrast, there is a lack of investigation into the ability of nursing clinical teachers to manage NSs suffering from WPV.

**Method:**

A cross-sectional survey was conducted in Hubei province, China, between June and July 2022. Convenience sampling was employed to recruit clinical teachers in 9 tertiary hospitals who met the inclusion criteria. The survey was conducted using a self-designed questionnaire which has good reliability and validity. Questionnaires were collected through a web-based platform called Questionnaire Star in China. In July 2022, a total of 900 questionnaires were eventually distributed, and 869 valid questionnaires were returned. The software SPSS 26.0 was used to conduct descriptive and inferential statistical analyses.

**Result:**

The mean ± SD knowledge, attitudes, and practices scores of the nursing clinical teachers were 69.51 ± 12.78, 29.11 ± 4.38, and 82.84 ± 13.60, respectively. Also, the average score rate was 77.24%, 83.17%, and 87.20%, respectively. There are still deficiencies in the knowledge, attitudes, and practices index, such as knowledge about the sources of violence, perceptions about influencing factors, and behavioural practices for prevention. Factors affecting the knowledge index scores included teachers' age, highest academic degree obtained, years of work, the highest level of education that they could teach NSs, whether they had experienced WPV, whether they had witnessed NSs suffering from WPV, and whether their NSs had suffered from WPV. Factors affecting scores on the attitude index included the age, highest academic degree obtained, years of work, the highest level of education that they could teach NSs, and whether they had received training on prevention and coping with WPV. Factors influencing the score for the practice index included the working department, highest academic degree obtained, professional title, the highest level of education that they could teach NSs, and whether they had received training on prevention and coping with WPV.

**Conclusion:**

Nursing clinical teachers showed moderate levels of knowledge, attitudes, and practices about NSs suffering from WPV. The deficiencies mainly focused on the sources, manifestations, influencing factors, and preventive strategies of it. Also, nursing clinical teachers should be trained to better prevent and cope with WPV suffered by NSs. At the same time, in order to better protect and teach NSs, the selection criteria for nursing clinical teachers should be stricter, with requirements on age, education, years of working experience, and titles. *Implications for Nursing Management*. With nursing clinical teachers as the training target, health organizations and institutions should actively develop training related to preventing and coping with NSs suffering from WPV. Also, it should be incorporated into the preservice training system for nursing clinical teachers. The content of training could focus on the sources, manifestations, influencing factors, and prevention strategies of NSs suffering from WPV. The method could be online theoretical teaching combined with offline scenario simulation exercises. At the same time, strict admission criteria for nursing clinical teachers can be helpful in protecting the safety of NSs during the clinical rotation phase. For nursing managers, they should be kept abreast of organisational policies and adopt a zero-tolerance policy towards violence. It also can be recommended that nursing clinical teachers should be proactively involved in training to improve their own ability to protect NSs.

## 1. Introduction

The COVID-19 emergency highlights the importance of nurses but also exacerbates the nursing workforce shortage. Nursing students (NSs) are the hope of the caring profession. Retention of them is a strategy to solve the problem [1]. Workplace violence (WPV), a worldwide problem [2, 3], has been shown to be one of the important factors affecting NSs' career choice satisfaction [4]. Therefore, it is important to help them in preventing and managing WPV. Nursing clinical teachers are good individuals to achieve the aim.

In clinical rotation, nursing clinical teachers are the people who have the most contact with NSs. It is one of their responsibilities to help NSs manage WPV. However, there is little existing research about it. This study aims to assess nursing clinical teachers' knowledge, attitudes, and practices toward NSs suffering from WPV.

## 2. Background

WHO defined WPV in 2002 as “incidents where staffs are abused, threatened or assaulted in circumstances related to their work, involving an explicit or implicit challenge to their safety, well-being or health” [5]. It includes physical assault, verbal abuse, threats, sexual harassment, and other forms [6]. Compared to any other medical workers, nurses were vulnerable to suffering from WPV [7]. However, the incidence of WPV experienced by NSs was also very high, ranging from 37.3% to 70% [8–10]. For NSs, the source of WPV includes patients, nursing clinical teachers, and other health workers and peers [3].

NSs who have been exposed to WPV may experience physical morbidities or have psychological symptoms, such as headache, anxiety, fear, and low self-confidence [8, 11, 12]. At the same time, WPV has the potential to affect their social life [13]. The more serious consequence is that WPV can lead to a significant decline in career choice satisfaction [4], creating further challenges for nursing, and exacerbating the workforce shortage [14]. Therefore, how to help NSs manage WPV needs to be given full attention.

In clinical rotation, nursing teachers are the most needed groups for NSs. They are important in teaching NSs the necessary professional knowledge and skills, including managing WPV. They are the best protectors of NSs, because of their extensive work experience and expertise. Actually, many WPVs cannot be easily detected, and the majority of NSs in response to WPV is silent [14]. Thus, nursing clinical teachers need to be aware of the emotional changes of NSs and to detect the development of negative emotions. At the same time, it is important to note that nursing clinical teachers can also be perpetrators of WPV to NSs [15]. So, it is necessary for teachers to learn about WPV and regulate their behaviour.

At present, most of the research on managing NSs suffering from WPV is focused on how to improve NSs' awareness and enhance their ability to prevent and cope with it [16, 17]. Only a few suggestions about nursing clinical teachers can be found in the discussion or implication of articles. MacDonald et al. have mentioned that nursing clinical teachers should consistently respect NSs in all interactions, regularly reflect on their own behaviour and communication, and establish facilitation skills to support and protect NSs as manage WPV [18]. It is necessary to strengthen the education or training programs to teach communication skills to nurses and nursing clinical teachers who participate in the clinical practicum of NSs [19]. At the same time, nursing clinical teachers by reviewing their past student experiences will also improve teaching methods and communication strategies [20]. These ideas can serve as a reminder of inspiration for the administrators or nursing clinical teachers, but not systematic. There is a paucity of evidence surrounding the extent to which nursing clinical teachers are formally prepared to respond to NSs suffering from WPV.

This study is guided by the theory of knowledge, attitudes, and practices (KAP theory). It is one of the common theories used to investigate the state of behavioural implementation and to explore the impact factor, including three elements: knowledge, attitudes, and practices [21]. Adequate knowledge helps to build positive attitudes, which are the basis for the emanation of practices. Attitudes are the driving force behind the development of practices [22]. Finding out about the level of clinical teachers' knowledge, attitudes, and practices regarding NSs suffering from WPV can help identify deficits in their managing. Based on the situation, measures can be taken to improve the level of knowledge to help them change the attitudes and enhance the ability to manage NSs suffering from WPV.

The aims of our study were to assess the knowledge, attitudes, and practices of nursing clinical teachers towards NSs suffering from WPV and to analyse the impact factors. We hope that this study can provide a foundation or a practical basis for developing a more comprehensive and scientific strategy or policy for the protection of NSs.

## 3. Methods

### 3.1. Design and Participants

A cross-sectional study was conducted in Hubei province, China, between June and July 2022. In this study, convenience sampling was employed to recruit nursing clinical teachers in 9 tertiary hospitals who met the inclusion criteria: (1) college or above education degree; (2) have obtained a certificate of nursing practice in China; (3) engaged in nursing clinical teaching for more than 1 year; (4) willingness to participate in this study and provide informed consent. The sample size should be 5 to 10 times the number of scale items [23]. At the same time, considering the sample dispersion rate of 20%, it was expected that at least 275 valid questionnaires need to be collected. Participants were encouraged to take part in online polls or finish offline surveys. A total of 900 questionnaires were eventually distributed.

### 3.2. Data Collection Instrument

We use a demographic information questionnaire and a self-designed scale. The demographic information included participants' gender, age, department, highest academic degree obtained, professional title, years of work, years of clinical teaching, and highest education level of NS who can be taught and four questions. Q1: Have you received training on prevention and coping with WPV? Q2: Have you ever experienced WPV? Q3: Have you ever witnessed WPV against NSs? Q4: Have your NSs been subjected to WPV?

The self-designed scale includes three sections.  Section 1 named “knowledge” revolved around the concept, sources, manifestations, consequences, and prevention and coping measures of NSs suffering from WPV and contained 5 second-level indexes and 18 items.  Section 2 named “attitudes” was set with the goal of exploring nursing clinical teachers' attitudes toward factors that influence the NSs suffering from WPV and contained 3 second-level indexes and 7 items.  Section 3 named “practices” shared 2 second-level indexes, prevention and coping, with 19 items. Each item was answered on a Likert 5-point scale ranging from strongly disagree (score 1) to strongly agree (score 5). Higher scores indicate higher levels of nursing clinical teachers' knowledge, attitudes, and practices about NSs suffering from WPV. The scale was developed in 2022. The first draft was formed through a literature review, semistructured interviews, and the Delphi method. We administered the questionnaire to 302 nursing clinical teachers from three hospitals in Hubei province, China. The collected data were subjected to item analysis, validity test, and reliability test. The item content validity index (I-CVI) of the scale's content validity fluctuated from 0.90 to 1.00, and the scale content validity index using the averaging method (S-CVI/Ave) was 0.981. The results of exploratory factor analysis (EFA) showed that there were 4 factors with eigenvalues greater than 1 in the knowledge index, with a cumulative variance contribution rate as high as 73.897%, and each item had a loading of >0.5 in only 1 of the factors. There were 2 factors for the attitudes index with a cumulative variance contribution of 75.388%, of which 1 item was deleted as its contribution to both factors was <0.5. The EFA was again performed, with a cumulative variance contribution of 81.046%, and the remaining items met the retention requirement. The number of factors for the practices index was 2, with a cumulative variance contribution of 82.052%, with each item loading >0.5 in only 1 factor. The structural validity of the scale was good. In terms of reliability, the Cronbach's *α* of the scale was 0.961, the Cronbach's *α* of the knowledge index was 0.920, the Cronbach's *α* of the attitudes index was 0.904, and the Cronbach's *α* of the practices index was 0.977 (*P* < 0.001). The intraclass correlation coefficient (ICC) of the scale was 0.650, and every index's ICCs were in the range of 0.740 to 0.881 (*P* < 0.001). The content of the scale is shown in Attachment 1.

### 3.3. Data Collection

We distributed and collected the questionnaire using a professional online questionnaire survey, evaluation, and voting platform called Questionnaire Star in China. In July 2022, 869 valid questionnaires were returned with a valid return rate of 96.56%.

### 3.4. Ethical Considerations

The study was approved by the Ethics Committee of Tongji Medical College of Huazhong University of Science and Technology (No. S124), and appropriate permission was obtained from the director of nursing of the participating hospitals. The questionnaire collection process is carried out on the premise that survey respondents participate in the survey voluntarily.

### 3.5. Data Analysis

SPSS 26.0 software was used for the analysis. Demographic information was described in the form of frequencies and percentages. As the data collected did not conform to a normal distribution, mean ± SD, the median (P_25_, P_75_), and average score rate (the average score/full score × 100%) were used to describe each index and item score. The Shapiro–Wilk test was performed, and the results showed that the data did not conform to a normal distribution. Therefore, the impact factors were analysed using nonparametric tests (Mann–Whitney *U* test and Kruskal–Wallis H test), and *P* < 0.05 indicates statistical significance. For the correlation analysis of the three index scores, Spearman's correlation analysis has been used.

## 4. Results

### 4.1. Characteristics of the Participants

A total of 869 nursing clinical teachers participated in this survey, of whom 848 (97.58%) were female and 21 (2.42%) were male; age ranges from 23 to 59 years, mean ± SD = 35.2 ± 5.9 years; 89.3% of the nursing clinical teachers had a maximum degree of bachelor's degree, and 61.91% had the title of supervising nurse practitioner. The other sociodemographic characteristics are shown in Table 1.

### 4.2. Questionnaire Scores

The average score rate of the knowledge, attitudes, and practices index was 77.24%, 83.17%, and 87.20%, respectively. In the knowledge index, the section on sources and forms of NSs suffering from WPV has a low average score rate of 62.25% and 74.05%; in the attitudes index, the nursing clinical teachers had lower recognition in the second-level index “NSs and patients”; in the practices index, nursing clinical teachers scored significantly worse on preventive behaviours than on coping behaviours as shown in Table 2.

### 4.3. Analysis of Impact Factors

The results of the single-factor analysis for each index are shown in Table 3. In the knowledge index, pairwise comparisons using the Tukey post hoc test showed that: (1) teachers younger than 40 scored much higher than those older than 50, while those younger than 30 scored higher than those in the 31–40 age group; (2) teachers with bachelor's degrees were significantly more knowledgeable than those with college degrees; (3) teachers with 6–10 and 11–15 years of working experience were significantly more knowledgeable than those with more than 15 years; (4) teachers with 1–5 years of clinical teaching experience were significantly more knowledgeable than those with more than 15 years; (5) in terms of the highest educational level of NSs that they can be taught, there was no significant difference in pairwise comparisons; (6) teachers who had experienced WPV were significantly more knowledgeable than those who had not; (7) teachers who had witnessed WPV against NSs were significantly more knowledgeable than those who had not; (8) teachers whose NSs had suffered from WPV had significantly higher levels of knowledge than teachers who had no such experience.

In the attitudes index, the result of pairwise comparisons showed that (1) teachers younger than 50 scored much higher than those older than 50; (2) teachers with bachelor's and master degrees scored significantly higher than those with college degrees; (3) teachers with more than 15 years of work scored significantly lower than those with 6–10 and 11–15 years of work; (4) as the highest educational level of NSs that can be taught increased, the scores of teachers increased gradually; (5) teachers who had received training on prevention and coping with WPV scored significantly higher than those who had not received training.

In the practices index, the result of pairwise comparisons showed that (1) the practical abilities of teachers working in the operating room were significantly higher than those of teachers working in the department of obstetrics and surgery; (2) in terms of teachers' highest academic degree obtained and the highest educational level of NSs that they can be taught, there was no significant difference in pairwise comparisons; (3) the practical abilities of teachers who were deputy chief nurses or above were significantly lower than that of teachers who were senior nurses and supervisor nurses; (4) the practical abilities of teachers who had received training on prevention and coping with WPV were significantly higher than those of teachers who had not received training.

### 4.4. Correlation analysis of the Three First-Level Indexes

There was a significant positive correlation between nursing clinical teachers' knowledge, attitudes, and practices towards NSs suffering from WPV (*P* < 0.05) as shown in Table 4.

## 5. Discussion

This study was the first to investigate WPV experienced by NSs from the perspective of nursing clinical teachers. The study was guided by the KAP theory. The findings revealed a significant correlation between the 3 indexes, proving the KAP theory's viability as the theoretical foundation and research framework for the study. From the results, it appears that the nursing clinical teachers who participated in the survey had a moderate level of knowledge, attitudes, and practices regarding WPV against NSs. Because WPV against NSs is a negative event with serious consequences, the nursing clinical teachers' knowledge, attitudes, and practices average score rate should be as close to 100% as possible. However, the average score rates of all indexes are not up to 90%. Therefore, the level is medium. In the knowledge index, the second-level indexes with the highest average score rate were “definition” and “consequences, prevention, and coping with WPV,” and the item with the highest average score rate was “it is nursing clinical teachers” duty to help NSs prevent WPV, indicating that the participating nursing clinical teachers have a strong responsibility and can be more aware of their role in protecting NSs. The second-level index with the lowest average score rate was “sources” of WPV for NSs. Further analysis of the contents of the three items with the lowest average score rate showed that they are not fully knowledgeable about the sources of WPV that NSs face. They were highly aware of external sources of WPV experienced by NSs (the perpetrators including patients and their families). But type III violence, which refers to violent acts by health worker to health worker, has not received enough attention and even lower awareness about the possibility of becoming perpetrators themselves. Therefore, nursing clinical teachers should be trained to increase their knowledge of WPV type III in NSs. In the attitudes index, the three lowest-scoring items all belong to the second-level index of “NSs and patients.” It can be demonstrated that they did not pay sufficient attention to NS factors and patient factors that may lead to NSs suffering from WPV, which may be related to insufficient teaching experience, lack of WPV training, or teachers' belief that nurses are unable to intervene in patients' behaviours and perceptions. Regarding practices, the abilities to prevent WPV experienced by NSs are inadequate. This is especially apparent in whether clinical teachers had learned the knowledge and skills to prevent and cope with WPV and the timing of invasive nursing practices chosen.

At the moment, hospitals and nursing management should take action to make up for the clinical teachers' lack of knowledge, attitudes, and practices. Only 48% of the clinical teachers had received training in managing WPV in the study. Also, the surveys have indicated that identified challenges in managing WPV include inadequate training resources and inconsistent training models [24]. As a result, it is essential that health organisations and institutions carry out training on the management of WPV in groups of clinical teachers, particularly about WPV that NSs may face. It can be included in the system of prejob training for them. In the theoretical education phase, web-based courses can be implemented to increase flexibility and participation in course scheduling. Also, according to the results of our study, whether a clinical nursing teacher has personally experienced WPV and whether one has witnessed and dealt with an NS being subjected to WPV will have an impact on the knowledge index scores. It can prove that the experience of WPV makes them more sensitive to the knowledge and more impressed with the content of education and training about it. Therefore, in behavioural training stages, scenario simulation rehearsal can also be added to the training to better help clinical nursing teachers achieve the transition from theory to practice. Khan et al. also have suggested that more realistic scenario-based interactions should be added to WPV management training for employees [25]. Additionally, the WPV-trained teachers performed better on the attitudes and practices indexes. This underscores the importance of teachers' active engagement in the WPV management training. Thus, clinical teachers also should have a positive mindset and take the initiative to participate in training and learning in parallel to health organisations and institutions carrying out training on the management of WPV. After learning about NSs suffering from WPV, they should consciously regulate teaching behaviour.

Nursing clinical teachers' knowledge and attitudes scores are influenced by their age and years of work. Those who are younger with 6–10 years' work and 11–15 years' work in clinical are more able to learn new knowledge, more adaptable, and more experienced and have a systematic clinical mindset. These advantages can help them to better undertake the task of clinical teaching. The highest academic degree obtained is also an influencing factor. In China, undergraduate education focuses more on the development of systematic thinking than specialist education. Also, it also places a higher emphasis on students' mastery of theoretical professional knowledge. Therefore, teachers with bachelor's degrees scored significantly higher in all three areas than teachers with college degrees. In conclusion, when choosing clinical teachers, these aspects should be taken into consideration. It might make it possible to teach and protect NSs in clinical rotation using a more comprehensive and scientific approach.

In the department, the management of NSs suffering WPV within the department is equally important. Leaders should manage NSs systematically and impartially. It is feasible to provide the necessary support to nursing clinical teachers and NSs, such as opening channels for reporting. At the same time, there should be a zero-tolerance attitude and management because it can effectively reduce the incidence of WPV [26]. Also, leaders' attitudes and approaches to violence resolution are critical in the link between WPV and victims' depressive symptoms [27], so it will also increase NSs' sense of organisational security.

## 6. Conclusions

In terms of managing WPV experienced by NSs, this study was the first to investigate from the perspective of nursing clinical teachers. In China, nursing clinical teachers in the knowledge, attitudes, and practices indexes of NSs suffer from WPV on medium. There are still deficiencies in knowledge about the sources of violence, perceptions about influencing factors, and behavioural practices for prevention. Therefore, due attention should be given to the training and education of clinical teachers in the management of NSs suffering from WPV. When selecting clinical teachers, the criteria of them need to be strictly controlled.

## 7. Implications for Nursing Management

NSs subjected to WPV should be given full attention. Before clinical nurses become teachers, it is necessary to develop targeted training around the management of WPV experienced by NSs, including the causes, sources, prevention, and coping with violence. Because of the busy work schedule, training can take the form of a combination of online theoretical teaching and offline scenario simulation practices. Department managers should provide the necessary internal organisational support for NSs and clinical teachers. At the same time, the admission criteria for teachers should be strict. Their age, experience of work, highest academic degree obtained, and professional competence should be considered whether being in the most suitable range for teaching work. These can help to improve the effectiveness of the management of NSs suffering from WPV.

## Figures and Tables

**Table 1:** Demographic details (*N* = 869).

Variables	*N*	%	Knowledge Mean ± SD	Attitude Mean ± SD	Practice Mean ± SD
*Gender*					
Female	848	97.58	69.45 ± 12.85	29.09 ± 4.40	82.80 ± 12.63
Male	21	2.42	72.05 ± 9.31	29.86 ± 3.53	84.24 ± 12.40

*Age*					
24–30	166	19.10	71.23 ± 13.46	29.61 ± 4.34	83.58 ± 13.02
31–40	553	63.64	69.08 ± 12.39	29.26 ± 4.34	83.27 ± 13.36
41–50	131	15.07	67.42 ± 12.94	28.34 ± 4.55	80.78 ± 14.79
>50	19	2.19	60.68 ± 12.42	25.79 ± 3.41	77.95 ± 15.63

*Department*					
Internal medicine	191	21.98	70.06 ± 12.01	29.13 ± 4.29	82.05 ± 13.66
Surgery	169	19.45	70.90 ± 12.88	29.41 ± 4.17	83.92 ± 12.91
Gynaecology	33	3.80	67.30 ± 12.69	28.79 ± 4.84	83.73 ± 13.51
Obstetrics	60	6.90	69.07 ± 13.39	29.92 ± 4.26	85.32 ± 12.49
Paediatrics	80	9.21	69.25 ± 12.74	28.68 ± 4.33	82.70 ± 13.48
General out-patient department	8	0.92	67.25 ± 17.04	26.50 ± 8.43	78.38 ± 28.70
Department for high fever	11	1.26	69.27 ± 10.97	29.64 ± 4.82	82.82 ± 13.23
Emergency	33	3.79	64.91 ± 13.19	28.36 ± 4.31	78.33 ± 14.45
ICU	50	5.75	69.96 ± 12.28	29.12 ± 3.50	84.86 ± 9.83
Operating room	37	4.26	64.46 ± 10.01	27.86 ± 3.46	74.30 ± 14.33
Infectious disease department	18	2.07	67.33 ± 15.08	27.33 ± 4.77	86.06 ± 10.18
Department of Psychiatry	9	1.04	75.44 ± 13.69	30.11 ± 4.76	85.11 ± 12.70
Other	170	19.56	70.14 ± 12.93	29.44 ± 4.67	83.55 ± 14.07

*The highest academic degree obtained*					
College degree	81	9.32	65.11 ± 14.05	27.00 ± 4.89	79.09 ± 15.64
Bachelor degree	776	89.30	69.93 ± 12.61	29.31 ± 4.27	83.27 ± 13.37
Master degree or above	12	1.38	72.64 ± 8.61	30.58 ± 3.75	80.50 ± 10.00

*Professional title*					
Nurse	7	0.81	65.43 ± 21.46	26.14 ± 5.67	74.71 ± 16.46
Senior nurse	265	30.49	69.18 ± 13.19	29,20 ± 4.41	82.19 ± 14.22
Supervisor nurse	538	61.91	70.19 ± 12.22	29.23 ± 4.29	83.85 ± 12.97
Deputy director nurse or above	59	6.79	65.36 ± 14.03	27.97 ± 4.66	77.49 ± 14.57

*Years of work*					
1–5	40	4.60	71.60 ± 9.20	28.87 ± 3.16	82.62 ± 11.97
6–10	289	33.26	70.84 ± 12.93	29.78 ± 4.21	83.61 ± 13.45
11–15	301	34.64	70.21 ± 12.14	29.24 ± 4.28	83.69 ± 12.77
>15	239	27.50	66.69 ± 13.49	28.18 ± 4.72	80.87 ± 14.86

*The duration of clinical teaching (year)*					
1–5	361	41.54	70.08 ± 12.78	29.12 ± 4.35	82.45 ± 13.63
6–10	305	35.10	70.25 ± 12.22	29.35 ± 4.27	83.72 ± 13.02
11–15	112	12.89	68.70 ± 13.06	29.34 ± 4.20	83.11 ± 14.14
>15	91	10.47	65.81 ± 13.73	28.01 ± 4.91	81.05 ± 14.64

*The highest educational level of NSs that can be taught*					
College degree	226	26.01	68.01 ± 12.63	28.15 ± 4.54	81.45 ± 13.57
Bachelor degree	587	67.55	69.87 ± 12.83	29.30 ± 4.30	83.14 ± 13.66
Master degree	56	6.44	71.91 ± 12.41	31.05 ± 3.66	85.30 ± 12.73

*Q1: Have you received training on prevention and coping with WPV?*					
Yes	421	48.45	69.45 ± 13.26	29.41 ± 4.58	83.84 ± 13.53
No	448	51.56	69.57 ± 12.33	28.84 ± 4.17	81.89 ± 13.61

*Q2: Have you ever experienced WPV?*					
Yes	462	53.16	70.71 ± 11.84	29.16 ± 4.09	82.69 ± 13.51
No	336	38.67	67.64 ± 14.05	28.90 ± 4.84	82.99 ± 13.87
Unsure	71	8.17	70.62 ± 11.37	29.83 ± 3.79	83.10 ± 13.03

*Q3: Have you ever witnessed WPV against NSs?*					
Yes	400	46.03	71.18 ± 11.54	29.12 ± 4.08	82.70 ± 13.20
No	396	45.57	67.99 ± 13.89	29.10 ± 4.76	83.02 ± 13.97
Unsure	73	8.40	68.62 ± 11.96	29.18 ± 3.79	82.58 ± 13.83
*Q4: Have your NSs been subjected to WPV?*					
Yes	234	26.93	72.34 ± 11.65	29.26 ± 4.16	82.80 ± 13.66
No	564	64.60	68.34 ± 13.11	29.09 ± 4.51	83.10 ± 13.49
Unsure	71	8.17	69.52 ± 12.33	28.82 ± 4.00	80.83 ± 14.22

**Table 2:** Score status.

Variables	Actual minimum scores	Actual maximum scores	Mean ± SD	M (P_25_, P_75_)	Average score rate (%)	Highest possible score
Knowledge index	18.00	90.00	69.51 ± 12.78	71.00 (62.00, 78.00)	77.24	90.00
Second-level indexes						
Definition	2.00	10.00	8.71 ± 1.64	9.00 (8.00, 10.00)	87.10	10.00
Source	4.00	20.00	12.45 ± 4.17	12.00 (10.00, 16.00)	62.25	20.00
Form	4.00	20.00	14.81 ± 5.22	16.00 (12.00, 20.00)	74.05	20.00
Consequence, prevention, and coping	8.00	40.00	33.54 ± 5.54	33.00 (31.00, 39.00)	83.85	40.00
Three lowest-scoring items						
K23	1.00	5.00	2.52 ± 1.34	2.00 (1.00, 4.00)	50.40	5.00
K24	1.00	5.00	2.88 ± 1.30	3.00 (2.00, 4.00)	57.60	5.00
K21	1.00	5.00	3.19 ± 1.20	3.00 (2.00, 4.00)	63.80	5.00
Attitudes index	7.00	35.00	29.11 ± 4.38	28.00 (27.00, 32.00)	83.17	35.00
Second-level indexes						
NSs and patients	4.00	20.00	16.15 ± 2.92	16.00 (15.00, 18.00)	80.75	20.00
Nursing clinical teachers	3.00	15.00	12.96 ± 2.00	12.00 (12.00, 15.00)	86.40	15.00
Three lowest-scoring items						
A12	1.00	5.00	3.94 ± 0.88	4.00 (4.00, 5.00)	78.80	5.00
A11	1.00	5.00	4.03 ± 0.84	4.00 (4.00, 5.00)	80.60	5.00
A13	1.00	5.00	4.05 ± 0.81	4.00 (4.00, 5.00)	81.00	5.00
Practices index	29.00	95.00	82.84 ± 13.60	87.00 (76.00,95.00)	87.20	95.00
Second-level indexes						
Prevention	11.00	55.00	47.50 ± 8.31	50.00 (44.00, 55.00)	86.36	55.00
Coping	8.00	40.00	35.33 ± 6.10	39.00 (32.00, 40.00)	88.33	40.00
Three lowest-scoring items						
P16	1.00	5.00	4.26 ± 0.89	4.00 (4.00, 5.00)	85.20	5.00
P11	1.00	5.00	4.27 ± 0.81	4.00 (4.00, 5.00)	85.40	5.00
P13	1.00	5.00	4.27 ± 0.84	4.00 (4.00, 5.00)	85.40	5.00

**Table 3:** Single-factor analysis of general demographic information for each index.

Variables	Knowledge index		Attitudes index		Practice index	
	*Z* or *H*^*∗∗*^	*P*	*Z* or *H*^*∗∗*^	*P*	*Z* or *H*^*∗∗*^	*P*
Gender	−0.802	0.422	−0.608	0.543	−0.275	0.783
Age	17.058	**0.001**	18.531	<**0.001**	7.298	0.063
Department	18.615	0.098	14.272	0.284	23.703	**0.022**
The highest academic degree obtained	9.501	**0.009**	22.796	<**0.001**	6.711	**0.035**
Professional title	7.069	0.070	7.494	0.058	12.829	**0.005**
Years of work	15.874	**0.001**	17.115	**0.001**	7.388	0.061
The duration of clinical teaching (year)	8.340	**0.039**	5.548	0.136	3.875	0.257
The highest educational level of NSs that can be taught	7.016	**0.030**	19.734	<**0.001**	7.200	**0.027**
Q1	−0.077	0.939	−2.410	**0.016**	−2.654	**0.008**
Q2	11.104	**0.004**	1.952	0.377	0.567	0.753
Q3	12.997	**0.002**	0.122	0.941	1.656	0.437
Q4	17.133	<**0.001**	1.196	0.550	1.848	0.397

^*∗∗*^For two independent samples, the Mann–Whitney *U* test for nonparametric tests was used, with the indicator being the *Z*-value. For multiple independent samples, the Kruskal–Wallis H test was used, reporting the H value of the test. Q1: Have you received training on prevention and coping with WPV? Q2: Have you ever experienced WPV? Q3: Have you ever witnessed WPV against NSs? Q4: Have your NSs been subjected to WPV? The bold values indicate *P* value less than 0.05.

**Table 4:** Correlation analysis of the three first-level indexes.

Variables	*r*	*P*
Knowledge—attitudes	0.531	<0.001
Knowledge—practices	0.421	<0.001
Attitudes—practices	0.615	<0.001

## Data Availability

Data are available upon request due to privacy/ethical restrictions.

## Supplementary Materials

Supplementary Materials The Attachment 1 is the self-designed knowledge, attitudes, and practices scale for nursing clinical teachers about student nurses suffering from workplace violence (English version) used by the study.

## References

[1] Middleton C. (2018). Morale in nursing students: a priority for nurse retention. *Journal of Advanced Nursing*.

[2] Tee S., Üzar Özçetin Y. S., Russell-Westhead M. (2016). Workplace violence experienced by nursing students: a UK survey. *Nurse Education Today*.

[3] Budden L. M., Birks M., Bagley T. (2017). Australian nursing students’ experience of bullying and/or harassment during clinical placement. *Collegian*.

[4] Furst C. (2018). The relationship between experiences of lateral violence and career choice satisfaction among nursing students. *Nursing Education Perspectives*.

[5] World Health Organization (2002). *Framework Guidelines for Addressing Workplace Violence in the Health Sector*.

[6] Boyle M. J., Wallis J. (2016). Working towards a definition for workplace violence actions in the health sector. *Safety & Health*.

[7] Huang L., Chang H., Peng X., Zhang F., Mo B., Liu Y. (2022). Formally reporting incidents of workplace violence among nurses: a scoping review. *Journal of Nursing Management*.

[8] Cheung K., Ching S. S., Cheng S. H. N., Ho S. S. M. (2019). Prevalence and impact of clinical violence towards nursing students in Hong Kong: a cross-sectional study. *BMJ Open*.

[9] Samadzadeh S., Aghamohammadi M. (2018). Violence against nursing students in the workplace: an iranian experience. *International Journal of Nursing Education Scholarship*.

[10] Pagnucci N., Ottonello G., Capponi D. (2022). Predictors of events of violence or aggression against nurses in the workplace: a scoping review. *Journal of Nursing Management*.

[11] Shdaifat E. A., Al Amer M. M., Jamama A. A. (2020). Verbal abuse and psychological disorders among nursing student interns in KSA. *Journal of Taibah University Medical Sciences*.

[12] Celebioglu A., Akpinar R. B., Kucukoglu S., Engin R. (2010). Violence experienced by Turkish nursing students in clinical settings: their emotions and behaviors. *Nurse Education Today*.

[13] Huang L., Wang Y., Huang H. (2021). Factors associated with family cohesion and adaptability among Chinese registered nurses. *Journal of Clinical Nursing*.

[14] Üzar-Özçetin Y. S., Russell-Westhead M., Tee S. (2021). Workplace violence: a qualitative study drawing on the perspectives of UK nursing students. *Collegian*.

[15] McKenna L., Boyle M. (2016). Midwifery student exposure to workplace violence in clinical settings: an exploratory study. *Nurse Education in Practice*.

[16] Gillespie G. L., Grubb P. L., Brown K., Boesch M. C., Ulrich D. (2017). Nurses eat their young: a novel bullying educational program for student nurses. *Journal of Nursing Education and Practice*.

[17] Brann M., Hartley D. (2017). Nursing student evaluation of NIOSH workplace violence prevention for nurses online course. *Journal of Safety Research*.

[18] MacDonald C. M., Hancock P. D., Kennedy D. M., MacDonald S. A., Watkins K. E., Baldwin D. D. (2022). Incivility in practice- incidence and experiences of nursing students in eastern Canada: a descriptive quantitative study. *Nurse Education Today*.

[19] Ahn Y., Choi J. (2019). Incivility experiences in clinical practicum education among nursing students. *Nurse Education Today*.

[20] Koharchik L. (2018). Nursing instructor incivility toward students. *AJN, American Journal of Nursing*.

[21] World Health Organization (2008). *Advocacy, Communication and Social Mobilization for TB Control: A Guide to Developing Knowledge, Attitude and Practice Surveys*.

[22] Xu N., Zhang Y., Zhang X. (2021). Knowledge, attitudes, and practices of urban residents toward COVID-19 in shaanxi during the post-lockdown period. *Frontiers in Public Health*.

[23] Li H., Zheng L., Le H. (2020). The mediating role of internalized stigma and shame on the relationship between COVID-19 related discrimination and mental health outcomes among back-to-school students in wuhan. *International Journal of Environmental Research and Public Health*.

[24] Queensland Health (2016). Occupational violence prevention in queensland health’s hospital and health services. https://www.health.qld.gov.au/__data/assets/pdf_file/0024/443265/occupational-violence-may2016.pdf.

[25] Khan M. N., Khan I., Ul-Haq Z. (2021). Managing violence against healthcare personnel in the emergency settings of Pakistan: a mixed methods study. *BMJ Open*.

[26] Burkley J. (2018). Adopt zero tolerance for hospital staff bullying nursing students. *AJN, American Journal of Nursing*.

[27] Kim J. H., Lee N., Kim J. Y., Kim S. J., Okechukwu C., Kim S. S. (2019). Organizational response to workplace violence, and its association with depressive symptoms: a nationwide survey of 1966 Korean EMS providers. *Journal of Occupational Health*.

