# Supplementary material for: Nursing Clinical Teachers' Knowledge, Attitudes, and Practices about Nursing Students Suffering from Workplace Violence in China: A Cross-Sectional Survey
Source: J Nurs Manag. 2023 Oct 16;2023:8844919. doi: 10.1155/2023/8844919 (PMC11919048; doi:10.1155/2023/8844919)
Supplement: Supplementary Materials — The Attachment 1 is the self-designed knowledge, attitudes, and practices scale for nursing clinical teachers about student nurses suffering from workplace violence (English version) used by the study. [file 8844919.f1.docx]

KAP Questionnaire for Nursing Clinical Teachers on Nursing Students Suffering from Workplace Violence

| **First-level Index** | **Second-level Index** | **Item** |
| --- | --- | --- |
| **KNOWLEDGE**  **（K）** | **K1**  **Definition**  **of WPV** | K1-1 Incidents, where staff is abused, threatened or assaulted in circumstances related to their work, were termed “**workplace violence**”. |
|  |  | K1-2 “**workplace violence**” involves an explicit or implicit challenge to victims’ safety, well-being or health. |
|  | **K2**  **Source of WPV** | K2-1 **Peers of nursing students** may violence against them in the workplace. |
|  |  | K2-2 **Patients or their caregivers** may violence against nursing students in the workplace. |
|  |  | K2-3 **Nursing clinical teachers** may violence against nursing students in the workplace. |
|  |  | K2-4 **Other staff** may violence against nursing students in the workplace. |
|  | **K3**  **Form of WPV** | K3-1 **Verbal abuse** against nursing students in the workplace, such as mocks, slurs, derogation of personal dignity, derogation of professional  position and so on. |
|  |  | K3-2 **Isolating** nursing students, **concealing, or blocking information** about nursing students, **ignoring** their **help-seeking**, and **rolling eyes out** against nursing students in the workplace. |
|  |  | K3-3 **Attacking** **out** against nursing students in the workplace, such as hitting, kicking, patting, tying, pushing, biting, throwing objects, wringing their arms, and dragging their hair. |
|  |  | K3-4 **Sexual harassment** against nursing students in the workplace, such as sexual pulling, hugging, kissing, or touching sensitive parts. |
|  | **K4**  **Consequence, prevention and coping with WPV** | K4-1 WPV enables nursing students to produce **physiological responses** such as headache and stomach pain, and even tissue damage. |
|  |  | K4-2 WPV makes nursing students fear, anxiety, reduced self-confidence and other **psychological reactions**. |
|  |  | K4-3 WPV influences nursing students' **career perceptions and (or) career choices**. |
|  |  | K4-4 Nursing students who have experienced peer workplace violence may **become the next to implement violence against their peers**. |
|  |  | K4-5 “Nursing students suffering from workplace violence” impacts the overall **development of the nursing profession**. |
|  |  | K4-6 “Nursing students suffering from workplace violence” **can be prevented scientifically and coped with reasonably**. |
|  |  | K4-7 **It is nursing clinical teachers' duty** to help nursing students prevent workplace violence. |
|  |  | K4-8 To some extent, **related organizations or departments** **in hospitals or schools** can help nursing students to prevent and cope with workplace violence. |
| **ATTITUDE**  **（A）** | **A1**  **Nursing Students and patients** | A1-1 I think whether nursing students will suffer from workplace violence is **related to their age, educational level, character, empathy, professional literacy, communication ability, etc**. |
|  |  | A1-2 I think whether nursing students will suffer from workplace violence is related to **their knowledge and mastery of the department environment and relevant professional knowledge.** |
|  |  | A1-3 I think whether nursing students can effectively prevent, identify and cope with workplace violence is related to **their mastery of knowledge about it.** |
|  |  | A1-4 I think whether nursing students will suffer from workplace violence is related to **the age, personality, occupation, educational level and condition of the patients they care for or their caregivers.** |
|  | **A2**  **Nursing Clinical Teachers** | A2-1 I think that **nursing clinical teachers with high quality, strong professional ability and rich clinical experience** can help nursing students reduce the risk of suffering from workplace violence. |
|  |  | A2-2 I think **nursing clinical teachers play an important role in helping nursing students prevent and cope with workplace violence**. |
|  |  | A2-3 I think clinical teachers **should master the knowledge and skills to prevent and deal with workplace violence**. |
| **PRACTICE（P）** | **P1**  **Prevention** | P1-1 I **learned the knowledge and skills** to prevent and cope with workplace violence. |
|  |  | P1-2 I **taught** nursing student**s** how to prevent, identify and respond to workplace violence that may occur in the department. |
|  |  | P1-3 I **proactively knew the personality characteristics and professional abilities** of s nursing students in charge and targeted efforts to help them prevent workplace violence. |
|  |  | P1-4 I helped nursing students **improve their language communication skills** to avoid workplace violence as much as possible. |
|  |  | P1-5 I helped nursing students **improve their clinical practice** to avoid workplace violence as much as possible. |
|  |  | P1-6 I **chose the right time** to allow nursing students to participate in invasive nursing operations to prevent workplace violence. |
|  |  | P1-7 I helped nursing students know **the personality, condition and other information of patients who may commit violence**, so as to prevent the occurrence of workplace violence. |
|  |  | P1-8 When there were **negative changes in the eyes, expression and tone of the patients or their caregivers,** I was keenly aware of and respond in time |
|  |  | P1-9 I **avoided transmitting negative emotions** to nursing students to prevent myself from violence against them. |
|  |  | P1-10 I **respected and protected** the privacy of nursing students. |
|  |  | P1-11 According to the **character and current state of my colleagues,** I reasonably arranged for nursing students to communicate with them. |
|  | **P2**  **Coping** | P2-1 When nursing students suffered from verbal violence, I **pacified the perpetrators' emotions in time** to avoid more serious consequences as far as possible. |
|  |  | P2-2 When the nursing students suffered physical violence, I **protected them and sought the help** of colleagues and the security department in time. |
|  |  | P2-3 When the situation worsened and tended to get out of control, I **made the correct judgments, called for help in time and reported to the relevant departments of the hospital**. |
|  |  | P2-4 I **deeply understood the course of the incident and analyzed the causes** after nursing students suffered from workplace violence. |
|  |  | P2-5 I **timely understood their physiological and psychological status** after nursing students suffered from workplace violence. |
|  |  | P2-6 I **comforted and enlightened** nursing students after they suffered workplace violence. |
|  |  | P2-7 I **applied for leave or referral to psychological counseling** for nursing students suffering from workplace violence when necessary. |
|  |  | P2-8 When dealing with workplace violence, I f**ully respected the wishes** of nursing students and **safeguarded their legitimate rights and interests through effective ways**, such as actively seeking the help of superior leaders, and timely contacting their school teachers or family members for assistance, and applying for legal aid, etc. |
